# Thread Depth Effects on Stability and Positional Accuracy in Guided Immediate Implants: A Randomized Controlled Trial

**DOI:** 10.1111/cid.70145

**Published:** 2026-04-13

**Authors:** Na‐Yoon Kwon, Ji‐Young Jung, Kyung‐A Ko, Seung‐Hyun Park, Franz Josef Strauss, Jung‐Seok Lee

**Affiliations:** ^1^ Department of Periodontology Research Institute for Periodontal Regeneration, College of Dentistry, Yonsei University Seoul South Korea; ^2^ Clinic of Reconstructive Dentistry, Center of Dental Medicine, University of Zurich Zurich Switzerland; ^3^ Faculty of Health Sciences Universidad Autonoma de Chile Santiago Chile

**Keywords:** immediate implant, implant stability, macro‐design, marginal bone level, positional accuracy, thread depth

## Abstract

**Objectives:**

To evaluate the influence of implant thread depth on implant stability and positional accuracy in fully guided immediate implant placement.

**Materials and Methods:**

Fifty‐four patients received a reference tapered (BLT), a standard‐thread (MST), or a deep‐thread implant (MDT). Fifty‐four implants were placed using fully guided surgery and evaluated over 1‐year follow‐up. Primary outcomes were implant stability (insertion torque, ISQ, ISV). Secondary outcomes included positional accuracy and marginal bone level (MBL) at 12 months.

**Results:**

Primary and longitudinal stability were comparable across groups. Insertion torque was highest for MDT implants, followed by MST and BLT implants (BLT: 35.28 ± 11.18; MST: 40.00 ± 11.88; MDT: 43.89 ± 10.92 Ncm; *p* = 0.062). A higher proportion of MDT implants achieved insertion torque ≥ 40 Ncm (MDT: 14/18; MST: 12/18; BLT: 8/18). Angular deviation differed among implant groups (MDT: 5.57° ± 3.71°; BLT: 4.73° ± 2.46°; MST: 3.58° ± 1.81°; *p* = 0.062). In subgroup analysis comparing implants differing only in thread depth (MST vs. MDT), angular deviation was significantly greater for MDT (*p* = 0.029), particularly in molar sites. At 12 months, MBL did not differ significantly among groups.

**Conclusions:**

In fully guided immediate implant placement, deeper‐thread implants showed higher insertion torque values but were associated with greater angular deviation, while longitudinal stability and marginal bone levels remained comparable. Deeper‐thread designs may be beneficial when enhanced primary stability is required, provided careful angulation control is ensured.

**Trial Registration:**

cris.nih.go.kr Identifier: KCT0009402; This clinical trial was not registered prior to participant recruitment and randomization

## 1 Introduction

Extensive research has focused on optimizing dental implant thread design, particularly thread shape, height, width, and pitch [1]. These features are known to affect primary stability, load distribution, and stress transmission at the bone–implant interface, especially in low‐density bone. However, much of the existing evidence is derived from in vitro research, including finite element analyses and synthetic bone models which may not adequately reflect clinical conditions [2, 3].

Among the limited in vivo data, studies conducted in healed ridges suggest that increased thread depths may enhance primary stability, as indicated by higher insertion torque and implant stability quotient (ISQ) values [4, 5]. Nonetheless, whether these findings translate to more complex clinical scenarios remains unclear.

As immediate implant placement has become increasingly popular [6, 7, 8], the relevance of implant macro‐design has gained greater attention. Immediate implant placement may shorten treatment time, but it also poses unique anatomical challenges related to extraction socket morphology, which can compromise both implant stability and positional accuracy [9, 10].

Although the biomechanical principles underlying thread design are well established, their clinical relevance in immediate implant scenarios remains underexplored. Most available studies focus on healed ridges or controlled experimental conditions, which do not replicate the complexity of extraction sockets. Prosthetically driven implant placement is essential for optimal restorative design, reduction of biological complications [11, 12], and achievement of stable long‐term esthetic and clinical outcomes.

While advances in digital planning and guided surgery have improved placement precision, they may also introduce angular or positional deviations, especially in anterior regions or sites with narrow alveolar bone anatomy [13].

To date, no clinical study has comprehensively evaluated the dual effect of thread depth on both primary stability and positional accuracy under immediate implant placement conditions. In addition, the long‐term biological consequences of deeper threads, including their influence on marginal bone levels (MBL), remain poorly understood. Therefore, the aim of this randomized controlled trial was to assess the effect of increased thread depth on (1) primary stability, (2) positional accuracy, and (3) MBL during and after immediate implant placement in challenging clinical scenarios, with a one‐year follow‐up period.

## 2 Materials and Methods

### 2.1 Study Design

This single‐blinded, randomized controlled clinical trial evaluated the effects of implant thread design on primary stability, positional accuracy, and marginal bone level (MBL) over a 12‐month period. Ethical approval was obtained from the Institutional Review Board of Yonsei University Dental Hospital (IRB No. 2‐2020‐0101), and the study was conducted in accordance with the Declaration of Helsinki and the International Council for Harmonisation Good Clinical Practice (ICH‐GCP) guideline. Although the trial was retrospectively registered at the Clinical Research Information Service (CRiS; KCT0009402) due to an administrative oversight, all clinical procedures and outcome assessments were conducted according to a predefined protocol established before the first patient enrollment, and no protocol modifications were made after study initiation. Patients were not involved in the study design or outcome selection. Three implant systems with a self‐tapping, tapered, and SLA surface were evaluated:

- Reference tapered implant (BLT): Thread depth 0.30 mm (∅4.8 mm); Bone Level Tapered (Straumann, Basel, Switzerland)
- Standard‐thread implant (MST): Thread depth 0.40 mm (∅4.8 mm); BLUEDIAMOND Regular Thread (Megagen, Daegu, South Korea)
- Deep‐thread implant (MDT): Identical macro‐ and micro‐design to MST, differing only in thread depth (0.65 mm; ∅4.8 mm); BLUEDIAMOND Deep Thread (Megagen)

Patients aged ≥ 19 years requiring immediate implant placement after tooth extraction were recruited from the Department of Periodontology, Yonsei University Dental Hospital. Inclusion criteria required extraction sockets suitable for immediate placement with bone quality sufficient to achieve ≥ 20 Ncm insertion torque. Exclusion criteria included heavy smoking (> 20/day), pregnancy or lactation, uncontrolled systemic diseases, recent cancer therapy, or antiresorptive medication use. All participants provided written informed consent prior to enrollment and were followed for 12 months unless early withdrawal or implant failure occurred. This study was reported in accordance with the CONSORT 2025 guidelines for randomized controlled trials.

### 2.2 Sample Size and Randomization

Sample size was calculated with G*Power 3.1 based on expected differences in ISQ values [14], assuming an effect size 0.65, α = 0.05, and power = 95%. Fourteen implants per group were required. To account for a potential 20% dropout rate, 18 implants per group were enrolled.

Block randomization was used to ensure balance allocation. Allocation concealment was achieved using sealed opaque envelopes, opened by the surgeon immediately before implant placement. Participants were blinded to group assignment.

### 2.3 Surgical Procedures

Pre‐extraction cone‐beam computed tomography (CBCT) data were merged with intraoral scans (Trios 4; 3Shape, Copenhagen, Denmark) to perform three‐dimensional virtual implant planning using dedicated software (R2GATE Planning; Megagen). Implants were planned in prosthetically driven positions. Implant diameter and length were selected to achieve apical and/or proximal engagement of native bone.

Surgical guides (non‐metal sleeve type) with tripod stabilization were fabricated using three‐dimensional printing. In cases where complete seating of the surgical guide could not be achieved due to limited mouth opening or guide instability, the surgeon could convert to a freehand approach to ensure patient safety and optimal implant positioning. Such cases were defined as protocol deviations and were excluded from positional accuracy analysis but retained in stability analyses under ITT principles.

**FIGURE 1:**
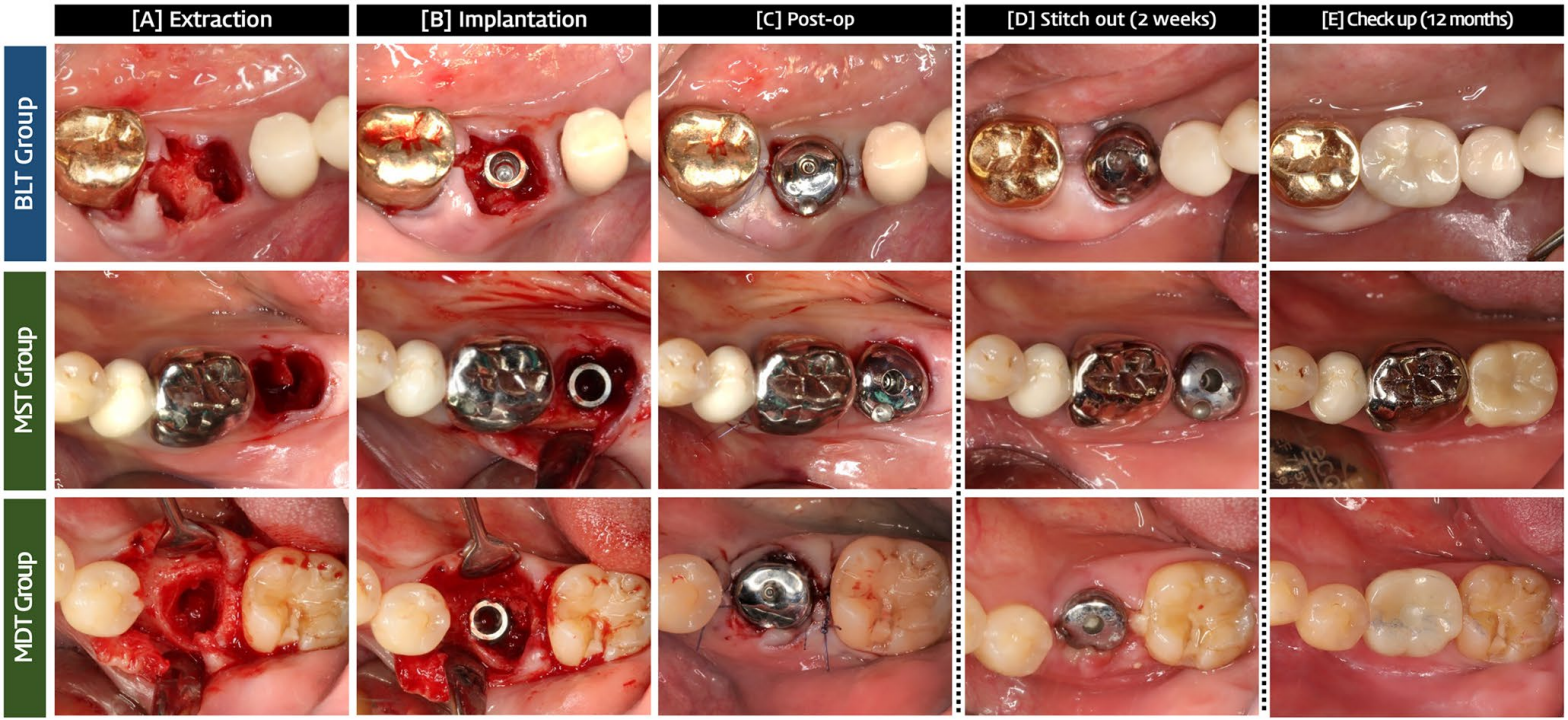
Clinical workflow of the study. (A) After atraumatic tooth extraction (B) after immediate implant placement (C) bone grafting or guided bone regeneration (GBR) was performed if needed. (D) Two weeks postoperative check‐up with stitch out (E) representative follow‐up photo of 12 months. Periodic follow‐ups were conducted at 1 month, 3 months (prosthesis delivery), 6 months, and 12 months.

Immediate implant placement was performed using a guided, flapless approach following tooth extraction. The overall surgical workflow is illustrated in Figure 1. When flap advancement was required, a sulcular incision with a vertical releasing incision was made. Socket management was standardized according to clinical criteria based on defect morphology observed after tooth extraction and implant placement.

Spontaneous healing was selected for intact extraction sockets with complete buccal and lingual plate integrity and horizontal gap distance ≤ 2 mm [15]. Gap defect management (grafting without membrane coverage) was performed when a horizontal jumping gap > 2 mm was present between the implant surface and socket wall, but without vertical dehiscence or loss of buccal plate continuity [16]. Guided bone regeneration (GBR) was performed when vertical or combined defects were identified, including buccal plate dehiscence ≥ 3 mm in height, absence of buccal wall support, or non‐contained defects requiring membrane stabilization. In such cases, a collagen membrane (BioGide; Geistlich, Switzerland) was applied to achieve defect containment. All socket management decisions were made according to these predefined criteria to minimize treatment heterogeneity.

Customized healing abutments were designed using CAD software (Exocad DentalCAD; Exocad GmbH, Germany) and milled from prefabricated titanium blocks (BX5 milling machine; Megagen). Abutments were designed to seal the socket entrance and were selectively modified in cases requiring flap advancement to optimize soft tissue margins. Customized healing abutments were connected, and transmucosal healing was achieved in all cases.

Final insertion torque was recorded intraoperatively. Implant stability was assessed using resonance frequency analysis (ISQ; Osstell AB, Sweden) and damping capacity analysis (ISV; Any‐Check, Neobiotech, Korea). Postoperative intraoral scans were obtained using scan bodies for positional accuracy analysis. Sutures (6–0 monofilament; Monosyn, B. Braun) were placed when flaps were raised and removed after 10–14 days.

### 2.4 Prosthetic Procedures

Prosthetic management followed standard clinical protocols according to implant site and functional requirements. In anterior sites, removable provisional dentures were used during the initial healing period when appropriate to avoid loading of the implants. Definitive restorations were delivered ~3 months after surgery. They were predominantly screw‐retained single crowns. However, in selected anterior cases where screw‐retained restoration was not feasible due to implant angulation or limited prosthetic space, cement‐retained crowns were provided. After delivery of the definitive prosthesis, removal was not routinely performed solely for research measurements in order to avoid unnecessary prosthetic complications and patient discomfort. In particular, removal of cement‐retained restorations or small anterior crowns was avoided when it was considered clinically unfavorable. Consequently, some resonance frequency analysis (ISQ) measurements could not be obtained at later follow‐up visits. These missing measurements reflect prosthetic workflow considerations rather than implant stability‐related events.

### 2.5 Outcome Measures

Primary outcomes were measures of implant stability, including insertion torque (intraoperative) and ISQ and ISV values obtained immediately after placement and at 1, 3, 6, and 12 months postoperatively.

Secondary outcomes included positional accuracy of implant placement and marginal bone level (MBL) at 12 months. Positional accuracy was assessed by superimposing presurgical planning data with postoperative intraoral scans using 3D inspection software (Geomagic Control X, 3D Systems, USA). Initial alignment and automatic best‐fit registration were performed using stable adjacent tooth surfaces as reference landmarks. The implant region and extraction socket area were excluded from the alignment process to minimize bias related to surgical site changes. Deviations at the platform, apex, vertical position (mm), and angulation (°) were measured. All measurements were performed by a calibrated examiner blinded to implant group allocation.

MBL was assessed on standardized periapical radiographs obtained 12 months after implant placement. MBL was defined as the vertical distance from the implant platform to the first bone‐to‐implant contact at the mesial and distal aspects. Measurements were calibrated using known implant length to correct for magnification. Negative values indicated bone levels coronal to the implant platform, whereas positive values indicated bone levels apical to the platform. MBL measurements represent absolute bone levels at the 12‐month follow‐up and were not calculated as changes from baseline radiographs. Measurements were performed in triplicate, and the mean value was used. All radiographic and 3D analyses were conducted by a single calibrated examiner (N.Y.K). Early implant failure was defined as lack of osseointegration confirmed clinically and radiographically before prosthetic loading.

### 2.6 Statistical Analysis

Statistical analyses were performed using SPSS version 25 (IBM Corp., Armonk, NY, USA). Data normality was assessed using the Shapiro–Wilk test, and homogeneity of variances using Levene's test. For normally distributed data with homogeneous variances, one‐way analysis of variance (ANOVA) was applied. Welch's ANOVA was used for normally distributed data with unequal variances, and the Kruskal–Wallis test for non‐normally distributed data.

Longitudinal changes in implant stability (ISQ and ISV) were analyzed using linear mixed‐effects models to account for repeated measurements and missing data. An interaction term between thread group and time was initially included (thread group × time). Because no significant interaction was observed, the final models were fitted without interaction terms (ISQ: *p* = 0.399 [ITT], *p* = 0.730 [PP]; ISV: *p* = 0.284 [ITT], *p* = 0.264 [PP]).

To specifically evaluate the effect of thread depth, a predefined subgroup analysis compared the standard‐thread and deep‐thread groups, which differed only in thread depth. Depending on data distribution, comparisons were performed using independent *t*‐tests or Mann–Whitney *U* tests. A 2‐sided *p*‐value < 0.05 was considered statistically significant.

## 3 Results

### 3.1 Demographic Results

Baseline demographics are summarized in Table 1. The mean age was 60.8 ± 12.84 years. Gender distribution was balanced, and 90.7% were non‐smokers. Fourteen percent of patients had controlled diabetes (HbA1c ≤ 6.8%). Most implants (63.0%) were placed in the mandible, especially in molar sites (53.7%). Bone graft usage was similar across groups and did not differ significantly among groups (*p* > 0.05).

**TABLE 1:** Patient demographic characteristics of the study population.

	BLT group (*n* = 18)	MST group (*n* = 18)	MDT group (*n* = 18)	Total
Sex				
Male	11 (61.1)	6 (33.3)	10 (55.6)	27 (50)
Female	7 (38.9)	12 (66.7)	8 (44.4)	27 (50)
Age	60.8 ± 11.63	62.3 ± 13.86	59.4 ± 13.50	60.8 ± 12.84
Smoking				
Non smoker	18 (100)	15 (83.3)	16 (88.9)	49 (90.7)
Current smoker	0 (0)	3 (16.7)	2 (11.1)	5 (9.3)
Diabetes				
On treatment	4 (22.2)	2 (11.1)	2 (11.1)	8 (14.8)
Never	14 (77.8)	16 (88.9)	16 (88.9)	46 (85.2)
Type of jaw				
Mx	7 (38.9)	8 (44.4)	5 (27.8)	20 (37.0)
Mn	11 (61.1)	10 (55.6)	13 (72.2)	34 (63.0)
Surgical site				
Anterior	3 (16.7)	2 (11.1)	4 (22.2)	9 (16.7)
Premolar	7 (38.9)	6 (33.3)	3 (16.7)	16 (29.6)
Molar	8 (44.4)	10 (55.6)	11 (61.1)	29 (53.7)
Extraction socket management				
Spontaneous healing	5 (27.8)	4 (22.2)	5 (27.8)	14 (25.9)
Gap defect	9 (50)	8 (44.4)	9 (50)	26 (48.1)
GBR	4 (22.2)	6 (33.3)	4 (22.2)	14 (25.9)

*Note:* All diabetes patients are well controlled status showing HbA1C ≤ 6.8%. All current smoker patients smoke less than 10 cigarettes/day. The number in parentheses is the percentage of each variable.

**FIGURE 2:**
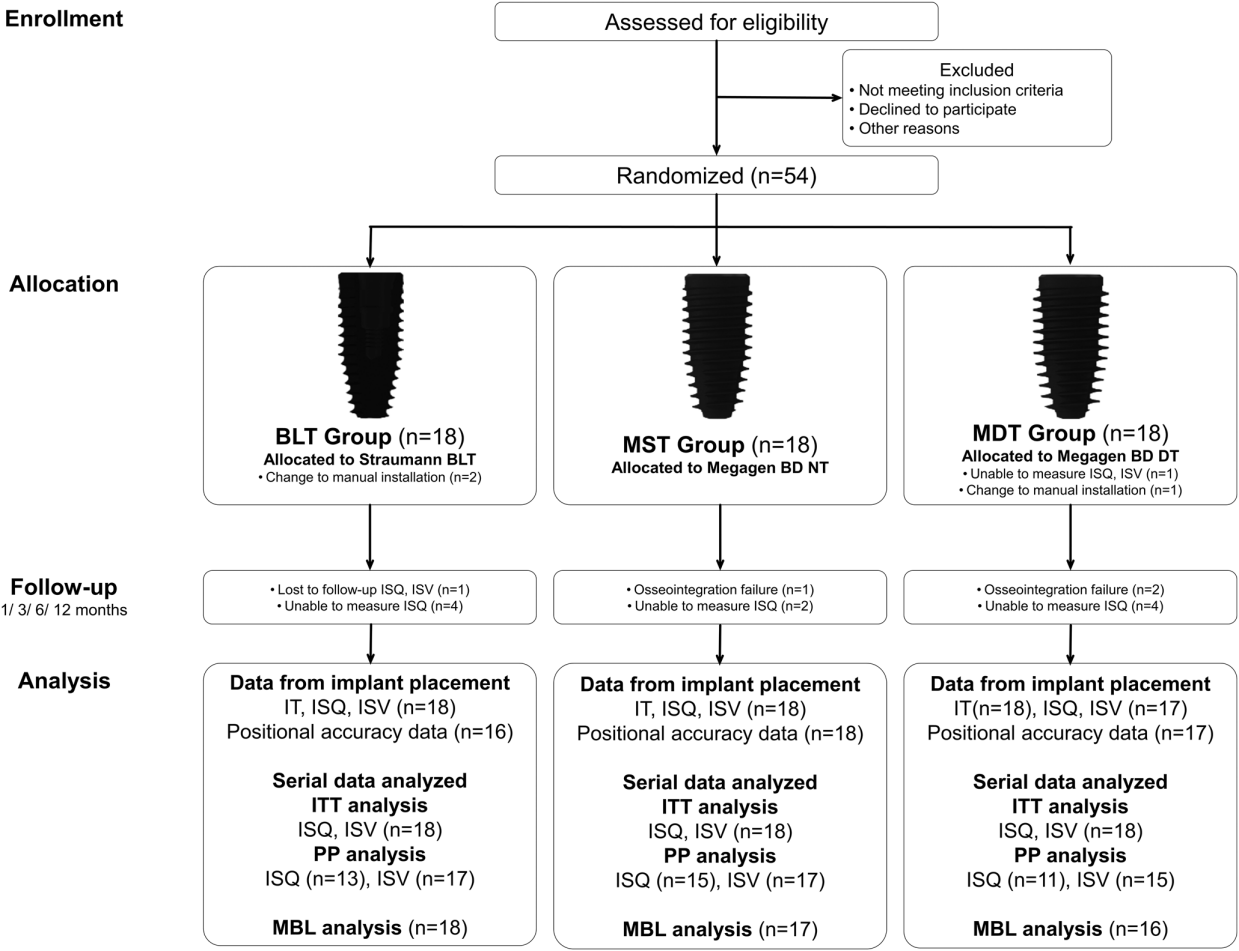
Study design flowchart.

A total of 54 patients were enrolled and randomized to three groups (BLT, MST, MDT; *n* = 18 each, Figure 2). The study was conducted from August 2021 to May 2025.

Baseline ISQ/ISV was not recorded for 1 MDT implant. Although the implant met the initial stability criterion (> 30 Ncm), its fixation relied primarily on a thin interradicular septum, which posed a risk of displacement during measurement. All other planned outcomes were collected. On participant in the BLT group was lost to follow‐up and excluded from follow‐up measurements. Three early implant failures occurred (MST: 1; MDT: 2). Serial ISQ measurements after prosthesis delivery were missing in 10 cases because the restoration could not be removed (BLT: 4; MST: 2; MDT: 4).

All 54 implants were included in intention‐to‐treat (ITT) analysis. For per‐protocol (PP) analysis of ISQ, ISV, and MBL, early failures and cases with missing measurements were excluded. In three cases (BLT: 2; MDT: 1), conversion to freehand placement was required due to intraoperative guide instability or insufficient seating caused by limited mouth opening or mechanical distortion. According to the study protocol, these cases were considered protocol deviations and excluded from positional accuracy analyses, but were retained in intention‐to‐treat analyses for stability outcomes.

### 3.2 Clinical Findings

All implants achieved an insertion torque of ≥ 20 Ncm and demonstrated clinical stability without rotational or pivotal movement during manual tightening. Transmucosal healing was uneventful, except for the 3 early failures. Minor postoperative symptoms such as swelling and pain resolved within 2 weeks. The peri‐implant mucosa healed without complications, and mucosal margin levels remained stable throughout the 12‐month follow‐up. No epithelial sloughing was observed during early healing, and the surrounding soft tissues provided adequate sealing around the customized healing abutment.

### 3.3 Implant Stability

**FIGURE 3:**
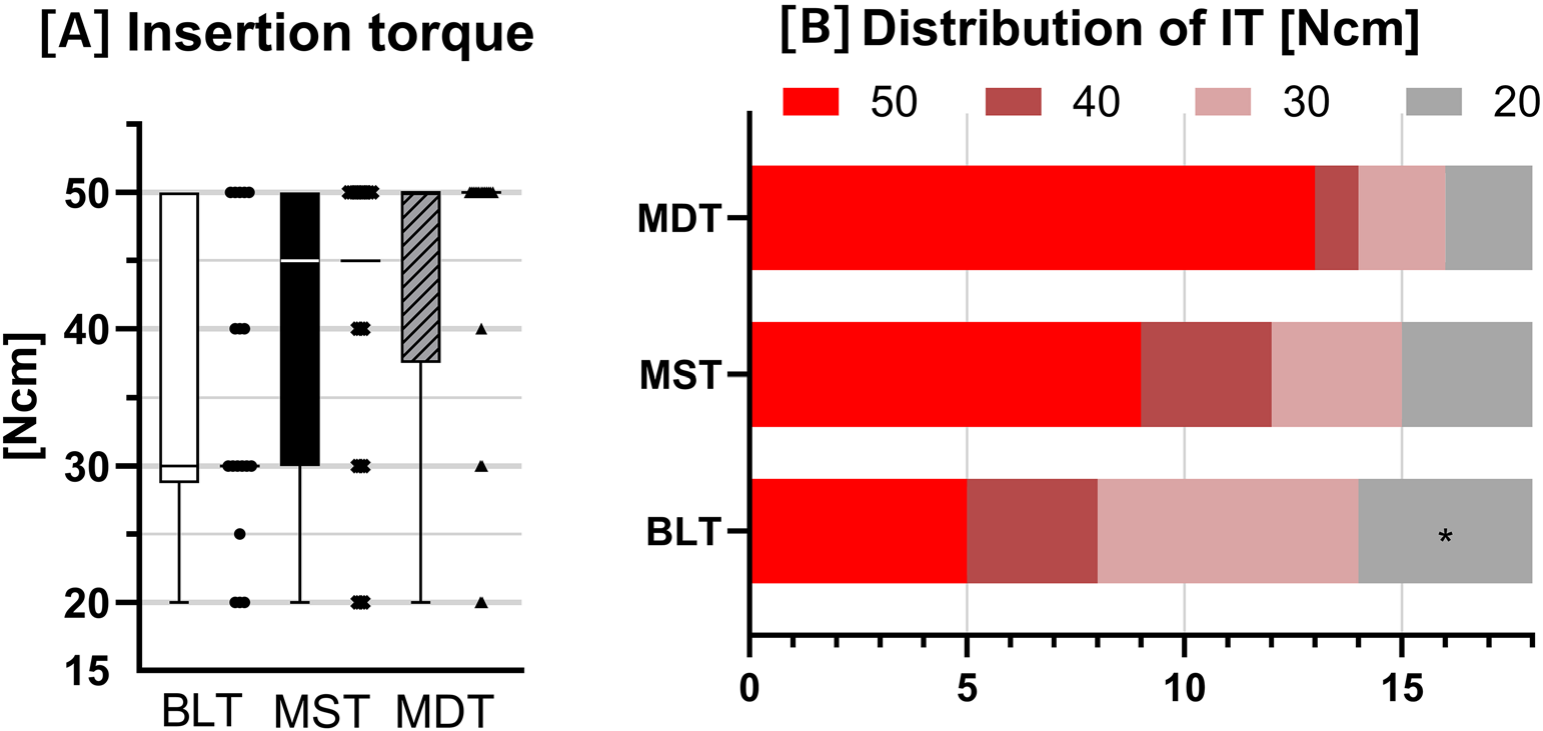
Box‐and‐scatter plots and stacked bar chart of insertion torque. (A) Box and scatter plots of insertion torque. (B) Stacked bar chart showing the distribution of insertion torque values, with MDT exhibiting a higher proportion at 50 Ncm.

The MDT group exhibited the highest mean insertion torque (43.89 ± 10.92 Ncm), followed by MST (40.00 ± 11.88 Ncm) and BLT (35.28 ± 11.18 Ncm). Although this difference approached statistical significance (*p* = 0.062), it represents an intergroup difference among implant systems. In the comparison of implants identical except for thread depth (MST vs. MDT), insertion torque was numerically higher in MDT. Analysis of insertion torque distribution further showed that, in the MDT group, all but five cases achieved 50 Ncm (Figure 3).

**FIGURE 4:**
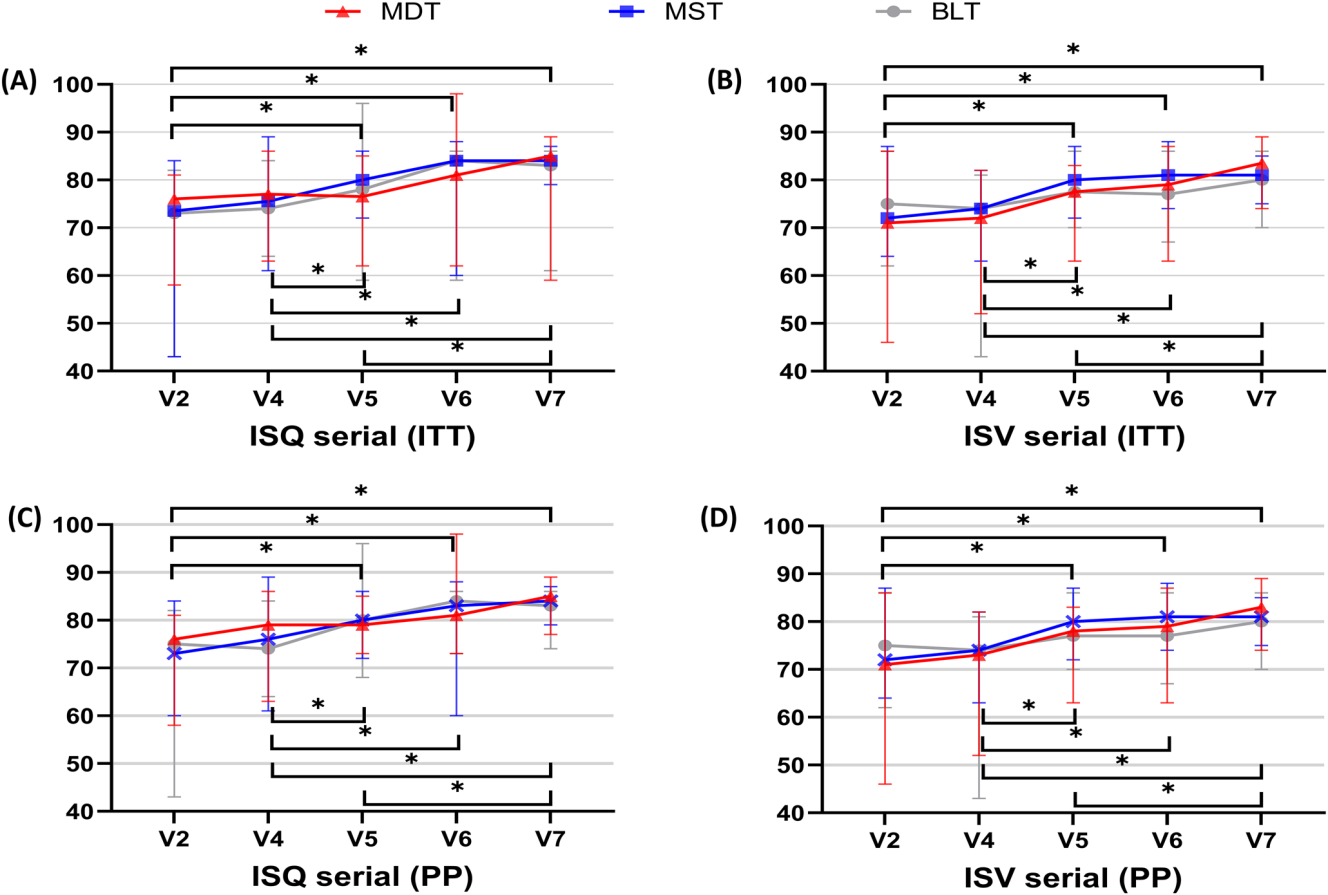
Median ISQ and ISV values across follow‐up visits.

Longitudinal implant stability (ISQ and ISV) analyzed using linear mixed‐effects models did not differ significantly among groups at any follow‐up point (immediately, and at 1, 3, 6, and 12 months after implant placement). Stability tended to increase consistently over time, across all thread designs, with the greatest gains observed between 1 and 3 months and between 3 and 12 months after implant placement (Figure 4). These findings were consistent in both ITT and PP analyses (Table 2).

**TABLE 2:** Implant stability over follow‐up time points (IT, ISQ, ISV).

		Implant stability								
		Implant placement (V2)	After 1 months (V4)		After 3 months (V5)		After 6 months (V6)		After 12 months (V7)	
			ITT	PP	ITT	PP	ITT	PP	ITT	PP
BLT	IT	35.28 ± 11.18	N‐A							
	ISQ	71.33 ± 9.31	73.81 ± 5.59	74.31 ± 5.94	77.56 ± 7.85	79.69 ± 6.95	81.00 ± 7.18	82.46 ± 4.10	80.94 ± 7.06	83.08 ± 3.30
	ISV	75.06 ± 6.65	71.83 ± 8.52	73.62 ± 4.99	78.56 ± 4.76	78.46 ± 4.50	77.06 ± 5.26	76.46 ± 4.22	80.29 ± 4.31	80.08 ± 4.59
MST	IT	40.00 ± 11.88	N‐A							
	ISQ	71.89 ± 9.37	74.56 ± 7.01	74.53 ± 7.26	79.82 ± 4.00	79.13 ± 3.72	79.12 ± 9.91	78.33 ± 10.31	83.75 ± 2.30	83.60 ± 2.29
	ISV	74.11 ± 5.93	73.71 ± 6.08	73.13 ± 6.08	78.88 ± 4.23	78.07 ± 3.71	80.94 ± 3.93	80.60 ± 4.07	81.12 ± 2.55	81.07 ± 2.66
MDT	IT	43.89 ± 10.92	N‐A							
	ISQ	73.88 ± 6.81	76.14 ± 6.79	76.91 ± 7.42	76.31 ± 7.03	79.55 ± 4.55	79.60 ± 8.48	82.36 ± 6.28	82.58 ± 8.20	84.73 ± 3.64
	ISV	71.53 ± 8.53	70.76 ± 9.02	72.91 ± 9.32	76.25 ± 5.27	78.91 ± 2.84	78.56 ± 7.29	79.73 ± 6.62	82.56 ± 5.27	83.09 ± 4.57
*p*	IT	0.062^‡^	N‐A							
	ISQ	0.70^‡^	0.61	0.79*	0.30*	0.96*	0.42^‡^	0.63^‡^	0.47^‡^	0.43^‡^
	ISV	0.41^‡^	0.65^‡^	0.51*	0.24*	0.96*	0.12^‡^	0.12^‡^	0.43^†^	0.39*

*Note:* Values are presented as average ± SD.

Abbreviations: ISQ, implant stability quotient; ISV, implant stability value; IT, insertion torque; ITT, intention‐to‐treat; PP, per‐protocol.

* *p*‐value for ANOVA.

^†^ *p*‐value for Welch's ANOVA test.

^‡^ *p*‐value for Kruskal‐Wallis test.

### 3.4 Positional Accuracy

MDT group showed the greatest angular deviation (5.57° ± 3.71°) compared to the other two groups (4.73° ± 2.46°, 3.58° ± 1.81° for BLT and MST group, respectively). Although overall intergroup differences approached statistical significance (*p* = 0.062), this represents variation among implant systems.

Subgroup analysis of matched implants (those differing only in thread depth, MST vs. MDT) showed that the MDT group had significantly greater angular deviation than the MST group (*p* = 0.029) indicating an effect of increased thread depth on angular accuracy. This effect was more pronounced when molar sites were analyzed separately. Notably, the four largest angular deviations (10.40°, 9.42°, 9.32°, and 9.20°), all occurring in mandibular molar sites, were observed exclusively in the MDT group.

**FIGURE 5:**
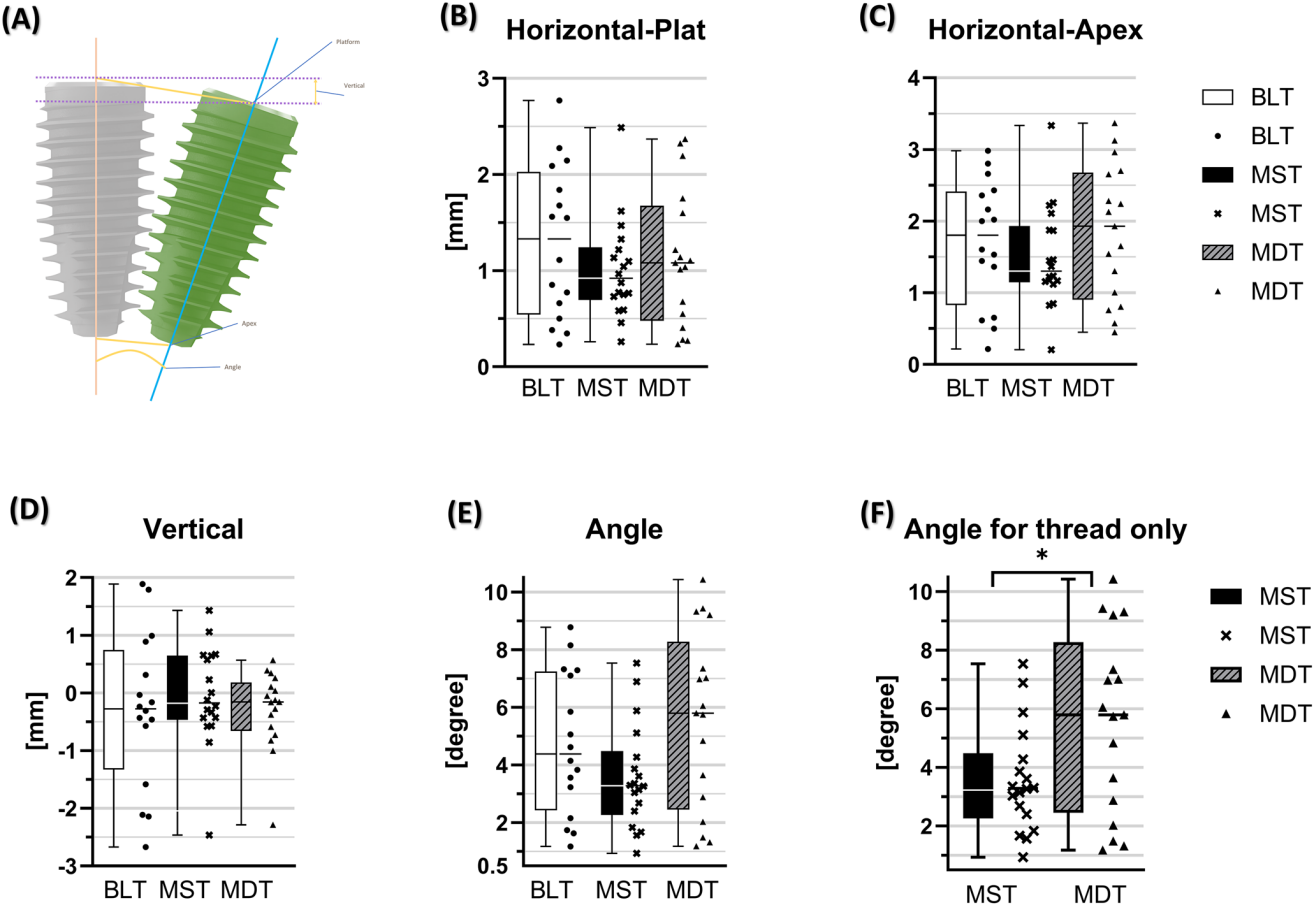
Schematic illustration and box‐and‐scatter plots for positional accuracy. (A) Schematic illustration of deviation measurements. (B–F) Box and scatter plots of positional accuracy.

Vertical deviation was lowest in MDT (0.49 ± 0.54 mm) and highest in BLT (1.04 ± 0.86), but this difference did not reach statistical significance (*p* = 0.12). Platform and apex deviations did not differ significantly across groups (*p* = 0.45 and 0.46, respectively) (Table 3 and Figure 5).

**TABLE 3:** Positional accuracy and marginal bone level measurements.

	Positional accuracy (V2)				Marginal bone level (V7)		
	Horizontal deviation (mm)		Vertical deviation (mm)	Angular deviation (degree)	Overall (mm)	Mesial (mm)	Distal (mm)
	Platform	Apex					
BLT	1.30 ± 0.80	1.71 ± 0.87	1.04 ± 0.86	4.73 ± 2.46	−0.40 ± 0.63	−0.56 ± 0.74	−0.24 ± 0.74
MST	1.01 ± 0.51	1.49 ± 0.70	0.64 ± 0.57	3.58 ± 1.81	−0.68 ± 0.84	−0.79 ± 1.05	−0.58 ± 0.95
MDT	1.13 ± 0.71	1.85 ± 0.94	0.49 ± 0.54	5.57 ± 3.71	−0.53 ± 0.85	−0.52 ± 1.03	−0.54 ± 0.93
*p*	0.46*	0.45*	0.12^‡^	0.062^†^	0.65^‡^	0.88^‡^	0.60*

*Note:* Values are presented as average ± SD.

* *p*‐value for ANOVA.

^†^ *p*‐value for Welch's ANOVA test.

^‡^ *p*‐value for Kruskal‐Wallis test.

### 3.5 Radiographic Analysis and MBL

**FIGURE 6:**
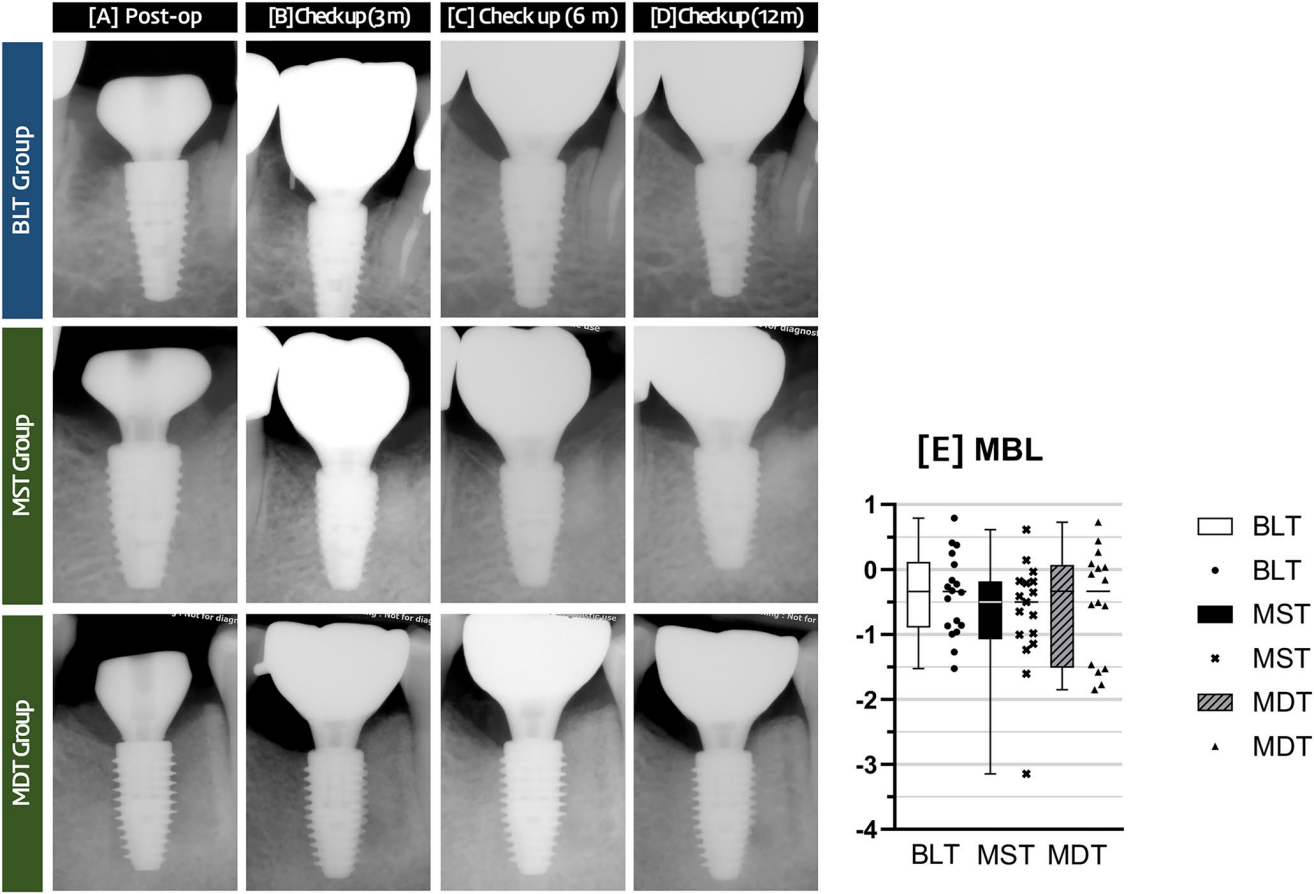
Periapical radiographs and box‐and‐scatter plots demonstrating marginal bone level. (A–D) Serially taken periapical radiographs. (E) Box and scatter plots of MBL at the 12‐month follow‐up.

At 12 months, overall MBL was −0.40 ± 0.63 mm (BLT), −0.53 ± 0.85 mm (MDT), and −0.68 ± 0.84 mm (MST), with no significant differences between the groups (*p* = 0.65). Bone levels at the 12‐month follow‐up were generally located coronal to the implant platform across all implant groups. No significant intergroup differences were detected for mesial (*p* = 0.88) or distal (*p* = 0.60) bone levels (Table 3 and Figure 6).

## 4 Discussion

This randomized clinical trial evaluated the combined effects of implant thread depth and guided surgery on primary and short‐term implant stability, positional accuracy and marginal bone stability in immediate implant placement. The main findings were as follows: (1) primary and longitudinal stability did not differ meaningfully among implant designs, although the deeper‐thread implants (MDT) showed a higher proportion of sites achieving elevated insertion torque thresholds (≥ 40 Ncm); (2) deeper‐thread implants (MDT) were associated with greater angular deviation, while overall placement accuracy remained within clinically acceptable limits across all sites; and (3) MBL remained stable over 12 months, with no differences attributable to thread depth.

All implants achieved insertion torque values within a clinically acceptable range (20–50 Ncm). Deeper‐thread implants showed a consistent tendency toward higher initial stability, reflected by a greater proportion of sites reaching elevated torque threshold (≥ 40 Ncm). This observation is consistent with biomechanical principles suggesting that increased thread depth enhances bone‐implant interlocking and thereby increases primary stability [17] as well as with previous studies in healed ridges [4, 5] or synthetic bone models [14].

The anatomical variability inherent to extraction sockets likely contributed to these findings. Immediate implant placement often involves irregular socket walls, limited apical engagement, and thin and inconsistent septal bone, all of which can influence insertion torque independently of implant design. In mandibular molar sites, initial stability may rely primarily on a narrow septum. In such situations, implants may be inadvertently displaced during seating, resulting in reduced initial torque despite appropriate drilling protocols and implant geometry [18]. These site‐specific challenges may limit the clinical expression of any theoretical mechanical advantage associated with deeper threads. Accordingly, in immediate implant placement, primary stability appears to be driven largely by local anatomy rather than thread depth alone.

Longitudinal stability, assessed using ISQ and ISV, increased steadily across all implant designs, indicating predictable osseointegration regardless of thread depth. The pronounced increase in stability between 1 and 3 months is in line with previously reported timelines for early bone remodeling [19, 20], and supports the feasibility of prosthetic loading at 3 months even under immediate placement protocols. The absence of differences among thread designs at any follow‐up interval suggests that once early healing is established, thread depth has limited influence on the trajectory of longer‐term implant stability.

Guided surgery yielded platform, apex, and angular deviations within clinically acceptable ranges for all three implant designs, comparable to values reported in previous meta‐analyses and clinical studies (entry deviation: ~1.2 mm; apex: ~1.5 mm; angular: 3°–5°) [9, 21, 22]. Importantly, earlier studies evaluated guided surgery in healed ridges, whereas the present trial examined fully guided placement in extraction sockets, where socket morphology may compromise placement accuracy. Experimental and cadaveric studies have demonstrated that early contact with cortical socket walls can deflect the implant from its planned trajectory [23, 24, 25]. The comparable overall positional accuracy across implant designs in the present study suggests that guided surgery may mitigate, at least in part, the anatomical variability inherent to immediate implant placement.

Despite similar overall accuracy, deeper‐thread implants demonstrated greater angular deviation than standard‐thread implant in the subgroup analysis where macro geometry was otherwise identical. This finding is clinically relevant because residual socket walls particularly the palatal/lingual walls or interradicular septal may redirect the implant during insertion. Deeper threads may engage these structures more aggressively, increasing susceptibility to off‐axis guidance and angular drift, particularly at low rotational drill speeds [13]. The occurrence of pronounced angular deviation (≥ 9°) exclusively in mandibular molar sites, in the MDT group highlights the for careful angulation control when using deeper‐thread implants in anatomically engagement with irregular socket walls. Three cases required intraoperative conversion to freehand placement due to guide instability or insufficient seating. These cases were excluded from positional accuracy analyses to avoid methodological bias. The occurrence of such conversions indicates that fully guided immediate implant placement may occasionally require intraoperative modification.

Increased angular deviation may have relevant prosthetic and biological implications. Even moderate deviations can alter the trajectory of screw access channels, require prosthetic compensation through angled abutments, or modify the restorative emergence profile. Particularly in posterior molar sites, where interradicular septal support is limited, small angular discrepancies may be amplified during implant seating and could affect restorative space management. Furthermore, excessive restorative emergence angles have been compromised peri‐implant tissue stability [26, 27, 28]. Although the angular deviations observed in the present study remained within ranges commonly reported for guided surgery and did not translate into detectable marginal bone changes at 12 months, the findings suggest that deeper‐thread designs may require heightened angulation control, especially in anatomically complex molar sites.

All implant thread designs demonstrated stable MBL at the 12‐month follow‐up. Because MBL were evaluated as absolute bone levels at the 12‐month examination, direct assessment of bone changes from implant placement was not performed. Stable MBL are well documented in healed ridges [29], whereas outcomes following immediate implant placement remain debated [7], with higher risks reported in damaged extraction sockets. In the present study, MBL remained well preserved regardless of thread depth or the use of simultaneous bone augmentation, consistent with a recent clinical trial [30]. Although micro thread configurations have been associated with favorable marginal bone maintenance [31], the current findings suggest that under a guided immediate placement protocol, thread depth alone does not meaningfully influence early marginal bone stability.

Early implant failures occurred in the MST (1) and MDT (2) groups, consistent with reports indicating higher early failure rates for immediate placement compared with healed sites [6, 7, 32]. Compromised socket conditions, including limited bone volume and damaged socket walls, are known to impair primary stability and increase the risk of early osseointegration failure [33, 34]. In the present study, early failures appeared more closely related to local anatomical conditions than to implant thread design, in line with recent observations [35, 36].

This study has limitations primarily related to the heterogeneity of the extraction sockets. Variability in socket damage, residual bone volume, and defect morphology may have influenced outcomes independently of implant design, limiting the ability to isolate the effect of thread depth. Although criteria were applied, managing for residual variability in socket morphology may still represent a potential confounding factor inherent to immediate implant placement studies. Future studies with stratified socket classifications or controlled defect models would further clarify the role of thread design in immediate implant placement.

The BLT implant was included as a well‐established implant system serving as a clinical reference comparator. Because BLT implants differ in macro‐design and manufacturer from MST and MDT implants, comparisons involving BLT implants reflect differences between implant systems rather than isolated effects of thread depth. Therefore, interpretation of thread depth effects should rely primarily on the matched comparison between MST and MDT implants, while the three‐arm comparison provides a broader clinical context and should be interpreted with caution.

## 5 Conclusion

Within the limitations of this study, implants with different thread depths demonstrated comparable primary and short‐term stability, as well as stable MBL, when placed using a fully guided immediate implant protocol. Although deeper‐thread implants exhibited a tendency toward higher insertion torque, they were also associated with greater angular deviation, particularly in molar sites. Deeper‐thread designs may therefore be considered as a potential option when enhanced primary stability is required, but their use in extraction sockets warrants careful attention to angulation control.

## Author Contributions

**Na‐Yoon Kwon:** data analysis, writing – original draft, visualization, writing – review and editing. Ji‐Young Jung: methodology, investigation, project administration. **Kyung‐A Ko:** validation, writing – review and editing. **Seung‐Hyun Park:** validation, writing – review and editing. **Franz Josef Strauss:** validation, writing – review and editing. **Jung‐Seok Lee:** conceptualization, methodology, investigation, writing – review and editing, supervision. All authors have read and agreed to the published version of the manuscript.

## Funding

This study was supported by a grant from MegaGen Implant Co., Ltd. and a grant from the National Research Foundation of Korea (NRF) funded by the Korea government (Ministry of Science and ICT, grant no. 2022R1A2C2005537).

## Ethics Statement

The study protocol was reviewed and approved by the Institutional Review Board of Yonsei University Dental Hospital, Seoul, Republic of Korea (IRB No. 2‐2020‐0101). The study was conducted in accordance with the Declaration of Helsinki and the International Council for Harmonisation Good Clinical Practice (ICH‐GCP) guidelines. All participants provided written informed consent prior to enrollment.

## Consent

Participants provided written informed consent for the publication of anonymized clinical data and associated images in scientific journals.

## Conflicts of Interest

The authors declare no conflicts of interest.

## Data Availability

The data that support the findings of this study are available from the corresponding author upon reasonable request.

## References

[1] H. Abuhussein , G. Pagni , A. Rebaudi , and H. L. Wang , “The Effect of Thread Pattern Upon Implant Osseointegration,” Clinical Oral Implants Research 21, no. 2 (2010): 129–136, 10.1111/j.1600-0501.2009.01800.x. 19709058

[2] O. Eraslan and O. Inan , “The Effect of Thread Design on Stress Distribution in a Solid Screw Implant: A 3D Finite Element Analysis,” Clinical Oral Investigations 14, no. 4 (2010): 411–416, 10.1007/s00784-009-0305-1. 19543925

[3] S. Fanali , M. Tumedei , P. Pignatelli , et al., “Comparative In Vitro Evaluation of the Primary Stability in D3 Synthetic Bone of Two Different Shapes and Pitches of the Implant Threads,” Applied Sciences 11, no. 12 (2021): 5612, 10.3390/app11125612.

[4] J. J. McCullough and P. R. Klokkevold , “The Effect of Implant Macro‐Thread Design on Implant Stability in the Early Post‐Operative Period: A Randomized, Controlled Pilot Study,” Clinical Oral Implants Research 28, no. 10 (2017): 1218–1226, 10.1111/clr.12945. 27699890

[5] M. Menini , F. Bagnasco , I. Calimodio , et al., “Influence of Implant Thread Morphology on Primary Stability: A Prospective Clinical Study,” BioMed Research International 2020 (2020): 6974050, 10.1155/2020/6974050. 32802868 PMC7426766

[6] J. Cosyn , L. De Lat , L. Seyssens , R. Doornewaard , E. Deschepper , and S. Vervaeke , “The Effectiveness of Immediate Implant Placement for Single Tooth Replacement Compared to Delayed Implant Placement: A Systematic Review and Meta‐Analysis,” Journal of Clinical Periodontology 46, no. Suppl 21 (2019): 224–241, 10.1111/jcpe.13054. 30624808

[7] M. S. Tonetti , R. E. Jung , G. Avila‐Ortiz , et al., “Management of the Extraction Socket and Timing of Implant Placement: Consensus Report and Clinical Recommendations of Group 3 of the XV European Workshop in Periodontology,” Journal of Clinical Periodontology 46, no. Suppl 21 (2019): 183–194, 10.1111/jcpe.13131. 31215112

[8] J. Pitman , V. Christiaens , J. Callens , et al., “Immediate Implant Placement With Flap or Flapless Surgery: A Systematic Review and Meta‐Analysis,” Journal of Clinical Periodontology 50, no. 6 (2023): 755–764, 10.1111/jcpe.13795. 36843361

[9] A. Tahmaseb , V. Wu , D. Wismeijer , W. Coucke , and C. Evans , “The Accuracy of Static Computer‐Aided Implant Surgery: A Systematic Review and Meta‐Analysis,” Clinical Oral Implants Research 29, no. Suppl 16 (2018): 416–435, 10.1111/clr.13346. 30328191

[10] T. Testori , T. Weinstein , F. Scutella , H. L. Wang , and G. Zucchelli , “Implant Placement in the Esthetic Area: Criteria for Positioning Single and Multiple Implants,” Periodontology 2000 77, no. 1 (2018): 176–196, 10.1111/prd.12211. 29484714

[11] D. Herrera , T. Berglundh , F. Schwarz , et al., “Prevention and Treatment of Peri‐Implant Diseases‐The EFP S3 Level Clinical Practice Guideline,” Journal of Clinical Periodontology 50, no. Suppl 26 (2023): 4–76, 10.1111/jcpe.13823. 37271498

[12] F. J. Strauss , J. Y. Park , J. S. Lee , et al., “Wide Restorative Emergence Angle Increases Marginal Bone Loss and Impairs Integrity of the Junctional Epithelium of the Implant Supracrestal Complex: A Preclinical Study,” Journal of Clinical Periodontology 51, no. 12 (2024): 1677–1687, 10.1111/jcpe.14070. 39385502 PMC11651719

[13] Y. W. Song , J. Y. Park , J. Y. Jung , J. N. Kim , K. S. Hu , and J. S. Lee , “Does the Fixture Thread Depth Affect the Accuracy of Implant Placement During Fully Guided Immediate Implant Placement?: A Human Cadaver Study,” Clinical Oral Implants Research 34, no. 2 (2023): 116–126, 10.1111/clr.14023. 36458928

[14] M. Karl and A. Irastorza‐Landa , “Does Implant Design Affect Primary Stability in Extraction Sites?,” Quintessence International 48, no. 3 (2017): 219–224, 10.3290/j.qi.a37690. 28168242

[15] D. Botticelli , T. Berglundh , and J. Lindhe , “Hard‐Tissue Alterations Following Immediate Implant Placement in Extraction Sites,” Journal of Clinical Periodontology 31, no. 10 (2004): 820–828, 10.1111/j.1600-051X.2004.00565.x. 15367183

[16] M. Sanz , J. Lindhe , J. Alcaraz , I. Sanz‐Sanchez , and D. Cecchinato , “The Effect of Placing a Bone Replacement Graft in the Gap at Immediately Placed Implants: A Randomized Clinical Trial,” Clinical Oral Implants Research 28, no. 8 (2017): 902–910, 10.1111/clr.12896. 27273298

[17] P. Trisi , M. Berardini , A. Falco , and M. Podaliri Vulpiani , “Effect of Implant Thread Geometry on Secondary Stability, Bone Density, and Bone‐To‐Implant Contact: A Biomechanical and Histological Analysis,” Implant Dentistry 24, no. 4 (2015): 384–391, 10.1097/ID.0000000000000269. 25939083

[18] H. Chen , W. Wang , and X. Gu , “Three‐Dimensional Alveolar Bone Assessment of Mandibular Molars for Immediate Implant Placement: A Virtual Implant Placement Study,” BMC Oral Health 21, no. 1 (2021): 478, 10.1186/s12903-021-01849-w. 34579702 PMC8474897

[19] M. A. Atieh , A. G. Payne , W. J. Duncan , R. K. de Silva , and M. P. Cullinan , “Immediate Placement or Immediate Restoration/Loading of Single Implants for Molar Tooth Replacement: A Systematic Review and Meta‐Analysis,” International Journal of Oral & Maxillofacial Implants 25, no. 2 (2010): 401–415. 20369102

[20] G. O. Gallucci , A. Hamilton , W. Zhou , D. Buser , and S. Chen , “Implant Placement and Loading Protocols in Partially Edentulous Patients: A Systematic Review,” Clinical Oral Implants Research 29, no. Suppl 16 (2018): 106–134, 10.1111/clr.13276. 30328194

[21] F. Bover‐Ramos , J. Viña‐Almunia , J. Cervera‐Ballester , M. Peñarrocha‐Diago , and B. García‐Mira , “Accuracy of Implant Placement With Computer‐Guided Surgery: A Systematic Review and Meta‐Analysis Comparing Cadaver, Clinical, and In Vitro Studies,” International Journal of Oral & Maxillofacial Implants 33, no. 1 (2018): 101–115, 10.11607/jomi.5556. 28632253

[22] J. Y. Park , Y. W. Song , S. H. Park , J. H. Kim , J. M. Park , and J. S. Lee , “Clinical Factors Influencing Implant Positioning by Guided Surgery Using a Nonmetal Sleeve Template in the Partially Edentulous Ridge: Multiple Regression Analysis of a Prospective Cohort,” Clinical Oral Implants Research 31, no. 12 (2020): 1187–1198, 10.1111/clr.13664. 32905643

[23] Z. Chen , J. Li , K. Sinjab , G. Mendonca , H. Yu , and H. L. Wang , “Accuracy of Flapless Immediate Implant Placement in Anterior Maxilla Using Computer‐Assisted Versus Freehand Surgery: A Cadaver Study,” Clinical Oral Implants Research 29, no. 12 (2018): 1186–1194, 10.1111/clr.13382. 30346631

[24] J. Li , P. C. Meneghetti , M. Galli , G. Mendonca , Z. Chen , and H. L. Wang , “Open‐Sleeve Templates for Computer‐Assisted Implant Surgery at Healed or Extraction Sockets: An In Vitro Comparison to Closed‐Sleeve Guided System and Free‐Hand Approach,” Clinical Oral Implants Research 33, no. 7 (2022): 757–767, 10.1111/clr.13957. 35578783

[25] G. Pruthi , R. Judge , J. Abduo , L. Ngo , and A. Gergely , “The Effect of Different Socket Morphologies of a Maxillary Central Incisor on the Accuracy of Immediate Implants Placed With Freehand or Guided Surgery‐An In Vitro Study,” Clinical Oral Implants Research 36, no. 6 (2025): 710–724, 10.1111/clr.14419. 39963729 PMC12146504

[26] G. Pelekos , B. Chin , X. Wu , M. R. Fok , J. Shi , and M. S. Tonetti , “Association of Crown Emergence Angle and Profile With Dental Plaque and Inflammation at Dental Implants,” Clinical Oral Implants Research 34, no. 10 (2023): 1047–1057, 10.1111/clr.14134. 37461128

[27] M. Pirc , J. Esquivel , A. Patrizzi , R. E. Jung , and F. J. Strauss , “Emergence Profile Angle Matters‐Restoring Peri‐Implant Health by Adjusting Prosthetics‐A Narrative Review,” Journal of Esthetic and Restorative Dentistry (2026), 10.1111/jerd.70109. PMC13193481 41618482

[28] M. Katafuchi , B. F. Weinstein , B. G. Leroux , Y. W. Chen , and D. M. Daubert , “Restoration Contour Is a Risk Indicator for Peri‐Implantitis: A Cross‐Sectional Radiographic Analysis,” Journal of Clinical Periodontology 45, no. 2 (2018): 225–232, 10.1111/jcpe.12829. 28985447

[29] T. Albrektsson , B. Chrcanovic , P. O. Ostman , and L. Sennerby , “Initial and Long‐Term Crestal Bone Responses to Modern Dental Implants,” Periodontology 2000 73, no. 1 (2017): 41–50, 10.1111/prd.12176. 28000272

[30] V. J. J. Donker , G. M. Raghoebar , K. W. Slagter , D. F. M. Hentenaar , A. Vissink , and H. J. A. Meijer , “Immediate Implant Placement With Immediate or Delayed Provisionalization in the Maxillary Aesthetic Zone: A 10‐Year Randomized Trial,” Journal of Clinical Periodontology 51, no. 6 (2024): 722–732, 10.1111/jcpe.13971. 38454548

[31] E. A. Bratu , M. Tandlich , and L. Shapira , “A Rough Surface Implant Neck With Microthreads Reduces the Amount of Marginal Bone Loss: A Prospective Clinical Study,” Clinical Oral Implants Research 20, no. 8 (2009): 827–832. 19508341 10.1111/j.1600-0501.2009.01730.x

[32] S. Mareque , P. Castelo‐Baz , J. Lopez‐Malla , J. Blanco , J. Nart , and C. Valles , “Clinical and Esthetic Outcomes of Immediate Implant Placement Compared to Alveolar Ridge Preservation: A Systematic Review and Meta‐Analysis,” Clinical Oral Investigations 25, no. 8 (2021): 4735–4748, 10.1007/s00784-021-03986-6. 34100157

[33] T. Urban , L. Kostopoulos , and A. Wenzel , “Immediate Implant Placement in Molar Regions: Risk Factors for Early Failure,” Clinical Oral Implants Research 23, no. 2 (2012): 220–227, 10.1111/j.1600-0501.2011.02167.x. 21457353

[34] V. I. Siciliano , G. E. Salvi , S. Matarasso , C. Cafiero , A. Blasi , and N. P. Lang , “Soft Tissues Healing at Immediate Transmucosal Implants Placed Into Molar Extraction Sites With Buccal Self‐Contained Dehiscences. A 12‐Month Controlled Clinical Trial,” Clinical Oral Implants Research 20, no. 5 (2009): 482–488, 10.1111/j.1600-0501.2008.01688.x. 19281503

[35] R. D. Wahlberg , V. F. Stenport , A. Wennerberg , and L. Hjalmarsson , “A Multicenter Study of Factors Related to Early Implant Failures‐Part 1: Implant Materials and Surgical Techniques,” Clinical Implant Dentistry and Related Research 27, no. 1 (2025): e70015, 10.1111/cid.70015. 39976277 PMC11840881

[36] R. D. Wahlberg , V. F. Stenport , A. Wennerberg , and L. Hjalmarsson , “A Multicenter Study of Factors Related to Early Implant Failures‐Part 2: Patient Factors,” Clinical Implant Dentistry and Related Research 27, no. 4 (2025): e70081, 10.1111/cid.70081. 40730516 PMC12307256

